# DEVELOPMENT OF TWO GERIATRIC CASES TO PREPARE STUDENTS FOR THE NEXT-GENERATION RN LICENSING EXAM

**DOI:** 10.1093/geroni/igad104.2113

**Published:** 2023-12-21

**Authors:** Mary DiBartolo

**Affiliations:** Salisbury University, Salisbury, Maryland, United States

## Abstract

With the implementation of the Next Generation NCLEX-RN (NGN) national licensing examination in April of 2023, faculty in state of Maryland nursing schools were solicited for participation in a Maryland Nursing Workforce Center grant initiative to develop a repository of NGN case studies. A major change in the exam was the integration of a six-step clinical judgment model using a case-based format with client information presented in an electronic medical record using new question types designated for each of the steps presented sequentially. Twenty faculty “champions” from 13 Maryland schools attended several training sessions and were to develop two peer-reviewed cases for inclusion in the Qualtrics database. Once approved, these cases could then be accessed online via a secure link or QR code by Maryland-based nursing faculty and shared with students for instructional and practice purposes. Two gero-focused cases were developed. The first case focused on an older adult in a long-term care facility admitted to the hospital for a urinary tract infection with potential complication of urosepsis. Another featured a hospitalized older adult client with Alzheimer’s disease admitted from home with dehydration with potential for delirium superimposed on dementia. While a major objective of this innovative and proactive state-wide initiative is to optimize success on the modified NCLEX-RN, it is also hoped that these case examples, when appropriately integrated into the nursing curriculum, will enhance understanding of common geriatric scenarios and better prepare graduates for safe, effective nursing care of older adults.

